# Differential Mechanisms of Action and Efficacy of Vitamin E Components in Antioxidant Cytoprotection of Human Retinal Pigment Epithelium

**DOI:** 10.3389/fphar.2021.798938

**Published:** 2022-01-04

**Authors:** R. Scott Duncan, Daniel T. Hurtado, Conner W. Hall, Peter Koulen

**Affiliations:** ^1^ Vision Research Center, Department of Ophthalmology, School of Medicine, University of Missouri—Kansas City, Kansas City, MO, United States; ^2^ Department of Biomedical Sciences, School of Medicine, University of Missouri—Kansas City, Kansas City, MO, United States

**Keywords:** retina, antioxidant, retinal pigment epithelium (RPE), age related macular degeneration (AMD), vitamin E, tocopherol, oxidative stress

## Abstract

The purpose of this study was to determine if different vitamin E components exhibit similar efficacy and mechanism of action in protecting Retinal pigment epithelium (RPE) cells from oxidative damage. We hypothesized that α-tocopherol (αT) is unique among vitamin E components in its cytoprotective mechanism of action against oxidative stress in RPE cells and that it requires protein synthesis for optimal antioxidant effect. We used cell viability assays, fluorescent chemical labeling of DNA and actin and immuno-labeling of the antioxidant proteins Nrf2 and Sod2 and of the tight junction protein, ZO-1, and confocal microscopy to determine the effects of αT and γT against oxidative stress in immortalized human RPE cells (hTERT-RPE). Using the four main vitamin E components, αT, γT, δ-tocopherol (δT) and α-tocotrienol (αTr), we ascertained that they exhibit similar, but not identical, antioxidant activity as αT when used at equimolar concentrations. In addition, we determined that the exposure time of RPE cells to α-tocopherol is critical for its ability to protect against oxidative damage. Lastly, we determined that αT, but not γT, partially requires the synthesis of new proteins within a 24-h period and prior to exposure to tBHP for optimal cytoprotection. We conclude that, unlike γT and δT, αT appears to be unique in its requirement for transport and/or signaling for it to be an effective antioxidant. As a result, more focus should be paid to which vitamin E components are used for antioxidant interventions.

## Introduction

Non-exudative or dry Age-Related Macular Degeneration (AMD), the most common form of AMD, is the leading cause of blindness in people over the age of 65 (Wong et al., 2014; Fleckenstein et al., 2021; Thomas et al., 2021). Dry AMD exhibits some genetic predisposition, but it is a multifactorial disease likely also requiring more than one therapeutic approach for treatment. Dry AMD pathophysiology includes Drusen deposition, oxidative stress, inflammation, geographic atrophy and, ultimately, vascular dysfunction (Hageman et al., 2001; Romero-Vazquez et al., 2021; Tisi et al., 2021; Toma et al., 2021). As a result, compounds that alter Drusen formation, reduce oxidative stress and inflammation, have the potential to prevent cellular loss and control of vascular changes can be potential therapeutic targets (Cabral de Guimaraes et al., 2021; Romero-Vazquez et al., 2021; Toma et al., 2021).

Oxidative stress is a major contributor to retinal pigment epithelium (RPE) dysfunction and cell death in dry AMD (Kunchithapautham et al., 2014; Marazita et al., 2016; Romero-Vazquez et al., 2021; Tisi et al., 2021; Toma et al., 2021). Common sources of oxidative stress in the retina include intrinsic factors such as mutations in complement factor genes, mitochondrial dysfunction and aging as well as extrinsic factors including excessive exposure to sunlight (blue light) and cigarette smoke (Hageman et al., 2001; Tomany et al., 2004; Edwards et al., 2005; Hageman et al., 2005; Khan et al., 2006; Seddon et al., 2006; Chakravarthy et al., 2007; Fletcher et al., 2008; He and Tombran-Tink, 2010; Kunchithapautham et al., 2014; Rohrer et al., 2016; Borras et al., 2019). Vitamin E has been studied as a potential therapy for diseases consisting of oxidative damage (Matsura, 2019; Cabral de Guimaraes et al., 2021). Vitamin E, and its constituent vitamers, exert at least some of their protective effects against oxidative stress through their direct free radical neutralizing effects, or as antioxidants. This is supported by extensive research over several decades (Sen et al., 2006; Traber and Atkinson, 2007; Zingg, 2019). More recently, however, it has become increasingly clear that tocopherols and tocotrienols can serve as signaling molecules to activate kinases and transcription factors alter gene expression (Chiricosta et al., 2019; Ghani et al., 2019; Gugliandolo et al., 2019; Mehrabi et al., 2019; Zingg, 2019; Hidalgo et al., 2020; Moore et al., 2020; Ding et al., 2021; Ungurianu et al., 2021; Willems et al., 2021).

The Age-Related Eye disease Study 1 (AREDS 1) clinical trial determined whether the oral delivery of nutritional supplements vitamin A, vitamin C, vitamin E, zinc and copper could improve outcomes in patients with wet and dry forms of AMD (Age-Related Eye Disease Study Research Group, 2001; Sackett and Schenning, 2002; SanGiovanni et al., 2008; Pemp et al., 2010; Chew et al., 2013). These supplements appeared to slightly reduce the risk for progression to wet AMD in some patients, but they had no effect on dry AMD. A follow-up study, AREDS 2, added omega-3 fatty acids and the carotenoids, lutein and zeaxanthin, but these additional compounds had no additional beneficial effect of patient outcomes compared to AREDS 1 (Age-Related Eye Disease Study 2 Research Group, 2013; Agrón et al., 2021). Overall, the studies suggested that vitamin, zinc and carotenoid supplementation alone is not effective in combating AMD.

There were limitations to the study design that did not adequately address whether vitamin E can exhibit therapeutic potential. One of the major problems with the AREDS studies was that the route of administration was oral, meaning that the supplements were subjected to first-pass metabolism leading likely to insufficient amounts reaching the affected tissues, namely the RPE (Hensley et al., 2004; Azzi, 2018; Uchida et al., 2018). First-pass metabolism in the liver changes the composition of vitamin E vitamers from predominately γ-tocopherol (γT) and other forms to primarily α-tocopherol (αT) (Azzi, 2018; Uchida et al., 2018). No clinical trial to date has tested whether alternative routes of vitamin E administration can improve clinical outcomes in dry AMD patients. Lastly, studying multiple test compounds or drugs at the same time in a mixture, or cocktail, resulting in complex pharmacokinetics and/or pharmacodynamics remains extremely challenging. With regard to vitamin E, it is reasonable to test individual tocopherols or tocotrienols or well-defined combinations thereof.

Vitamin E concentrations in blood serum has been determined in previous studies, but the concentrations in the retina obtained from normal dietary intake is not precisely known (Müller and Pallauf, 2003; Arigony et al., 2013; Azzi, 2018; Jamro et al., 2019; Zingg, 2019). Studies using animals such as rodents, canine and bovine sources indicate that the serum concentration of supplemented tocopherols, particularly αT, is in the low micromolar range (Müller and Pallauf, 2003; Jamro et al., 2019). As alluded to earlier, clinical studies utilizing vitamin E are difficult to interpret as γT and δT and tocotrienols are metabolized rapidly leaving only elevated levels of Αt (Hosomi et al., 2019).

The central nervous system, including the retina, has a high oxygen demand and, therefore, has an elevated rate of respiration leading to increased generation of reactive oxygen species from mitochondria (Liu et al., 2006; Rohrer et al., 2016). Furthermore, blue light can cause free radical formation in photoreceptor outer segments possibly leading to oxidative stress (Dalvi et al., 2019; Zhang et al., 2019). Vitamin E can reduce of polyunsaturated fatty acid auto-oxidation in the membranes of photoreceptor outer segments (Robison et al., 1982).

This study, to our knowledge, is the first of its kind in determining the comparative kinetics and cytoprotective efficacy between αT and γT against oxidative damage, the effects of these tocopherols on the expression and subcellular localization of antioxidant and structural proteins, and the requirement for protein synthesis for these observed effects in RPE cells.

Here, we determined whether specific components of vitamin E exhibit unique cytoprotective properties in immortalized human RPE cells (hTERT-RPE cells). We used supra-physiological concentrations (>25 μM) of γT, δT and αTr to ascertain whether they elicit similar cytoprotective effects as αT (Azzi, 2018). In addition, we determined whether short-term (4-h) versus longer-term (24-h) exposure to tocopherols results in a similar degree of cytoprotection against oxidative stress and cell death.

As cell signaling events often lead to changes in gene expression, and subsequent protein expression, we also determined whether αT and γT affect the expression of the antioxidant proteins, nuclear factor erythroid 2-related factor 2 (Nrf2) and superoxide dismutase 2 (Sod2) and the structural proteins, actin and zona occludens (ZO-1). In addition, we determined whether exposure of hTERT-RPE cells to tocopherols requires the synthesis of new proteins within a 24-h period and prior to exposure to tBHP for cytoprotection. If synthesis of new proteins by tocopherol-mediated cellular signaling is required for some or all their cytoprotective effects, this will provide clearer evidence of the importance of the signaling function of tocopherols, in addition to their direct antioxidant function, and shed light on new potential therapeutic targets.

## Materials and Methods

### Cell Culture and Treatments

Human telomerase reverse transcriptase-overexpressing RPE (hTERT-RPE) cells were provided by American Type Culture Collection (ATCC, # CRL-4000) and were maintained according to ATCC instructions. In brief, cells were grown in DMEM:F12 (1:1) + 10% FBS +10 μg/ml gentamicin to >50% density before splitting for growth or to full confluence for use in experiments. Tocopherols were obtained from Sigma-Aldrich (Millipore-Sigma, Burlington, MA, United States). Tocopherols were diluted in cell culture-grade DMSO to a working stock solution concentration of 100 mM and diluted in cell culture media to a final concentration of 100 μM. Tocopherol exposure times were either 4 h or 24 h prior to treatment with oxidant. Tert-butyl hydroperoxide (tBHP) (70% dilution, Acros Organics, Antwerp, Belgium), the oxidant used in this study, was diluted in ultrapure water (>18 Ω ohm·ml^−1^) to a final working concentration of 100 mM. The tBHP was diluted in culture media to a final concentration of between 100–500 μM for oxidative stress of cells. Cycloheximide (CHX) (Millipore Sigma, Burlington, MA, United States), used to inhibit protein synthesis, was diluted in PBS to a stock solution of 10 mM. CHX was diluted in media at a final concentration of 1 μM (IC50∼0.53 μM) and applied to cell 1 h prior to addition of tocopherols. A vehicle control (VC) was used as a control for each chemical or compound treatment. Experiments were carried out in triplicate.

### Calcein-AM Assay and Cell Staining

The calcein-AM assay was used to measure cellular esterase activity, a surrogate of cell viability. Calcein-AM dye (Invitrogen/Thermo Fisher Scientific, Waltham, MA, United States) was suspended in cell culture-grade DMSO to a final stock concentration of 2 mM and diluted in media to final concentration of 2–5 μM. The assay was carried out in detail as described elsewhere (Duncan et al., 2007). In brief, cellular loading of calcein-AM was carried out in Dulbecco’s PBS (Millipore Sigma) for 20 min. Plates were read at 485 nm/535 nm (excitation/emission) in a Flex Station three multi-mode plate reader (Molecular Devices). Detector sensitivity in plate reader assays was determined automatically by the instrument. Changes in calcein fluorescence in all experimental groups were compared to fluorescence values for the control groups.

An alternative measure of calcein fluorescence was conducted by observing calcein-AM dye-loaded cells from each condition under a fluorescence microscope. The camera exposure time settings were kept constant throughout image acquisition so that relative fluorescence intensities of cells and cellular morphology could be calculated from images. This approach was included to address high levels of calcein fluorescence in attached damaged cells that may give false positive readings in a plate reader assay.

For cell staining of fixed cells, nuclei were labeled with dilute 4′,6-diamidine-2′-phenylindole dihydrochloride (DAPI) stain (Millipore Sigma) and fluorescent dye 594 nm-labeled phalloidin (phalloidin-594; Abnova, Taipei City, Taiwan). Phalloidin labels only filamentous actin; non-filamentous, or globular, actin was not labeled. For DAPI and phalloidin labeling, cells were rinsed with DPBS and fixed for 20 min in 4% paraformaldehyde (PFA). After rinsing, PBS containing 0.5 μg/ml DAPI and 1:1,000 dilution of phalloidin-594 was added to cells followed by incubation for 2 h. Cells were then rinsed three times with PBS and mounted onto glass slides with Aqua Polymount medium (Polysciences, Warrington, PA, United States).

### Immunocytochemistry

Immunocytochemistry on treated cells was carried out as described elsewhere (Duncan et al., 2007). In brief, treated cells were rinsed with PBS, fixed with 4% paraformaldehyde for 20 min and then rinsed three times each for 5 min. After blocking, antibodies were applied to fixed cells overnight at 4°C. Antibodies used were mouse anti-Nrf2 (Abcam #ab89443) at 1:200, mouse anti-ZO-1 (Invitrogen #33-9100) at 1:200 and mouse anti-Sod2 (Invitrogen #A21990) at 1:250. Alexafluor™488- or Alexafluor™594-conjugated goat anti-rabbit or mouse IgG secondary antibodies (Invitrogen) were used for fluorescent labeling of cells. Coverslips were mounted using Aqua Polymount medium (Polysciences).

### Microscopy

Microscope images were acquired using a Leica TCS SP5-X white light laser scanning confocal microscope (Leica Microsystems, Mannheim, Germany) with fully motorized stage and CTR 6500 controller. DAPI and phalloidin labeling was detected using a diode laser (405 nm) with a detection range of 410–470 nm and tunable white light laser set at 590 nm excitation with a detection range of 600–650 nm. All images were acquired using 40X or 63X oil objectives and images were saved as Leica LIF files for later analysis.

### Image Analysis

Images were analyzed using Image-J FIJI software (open source, NIH, Bethesda, MD, United States). Images were converted to maximum intensity projection images in eight- or 16-bit TIFF format. Images were thresholded to remove background fluorescence leaving signal specific for the protein of interest. Mean and maximum grey level (intensity), area, % area and integrated density were calculated. Where necessary, histogram analyses were carried out to determine the counts at each pixel intensity. A cell counter tool in FIJI was used to manually count cell nuclei. To measure nuclear Nrf2 levels, colocalization analysis was conducted using the FIJI coloc2 tool.

### Statistics

Statistical difference between more than two groups, which includes all of the data herein, was carried out using a one-way ANOVA with a post hoc Bonferroni correction using GraphPad Prism software (GraphPad, San Diego, CA, United States).

## Results

### Sensitivity of hTERT-RPE Cells to tBHP-Mediated Oxidative Stress Damage

**FIGURE 1:**
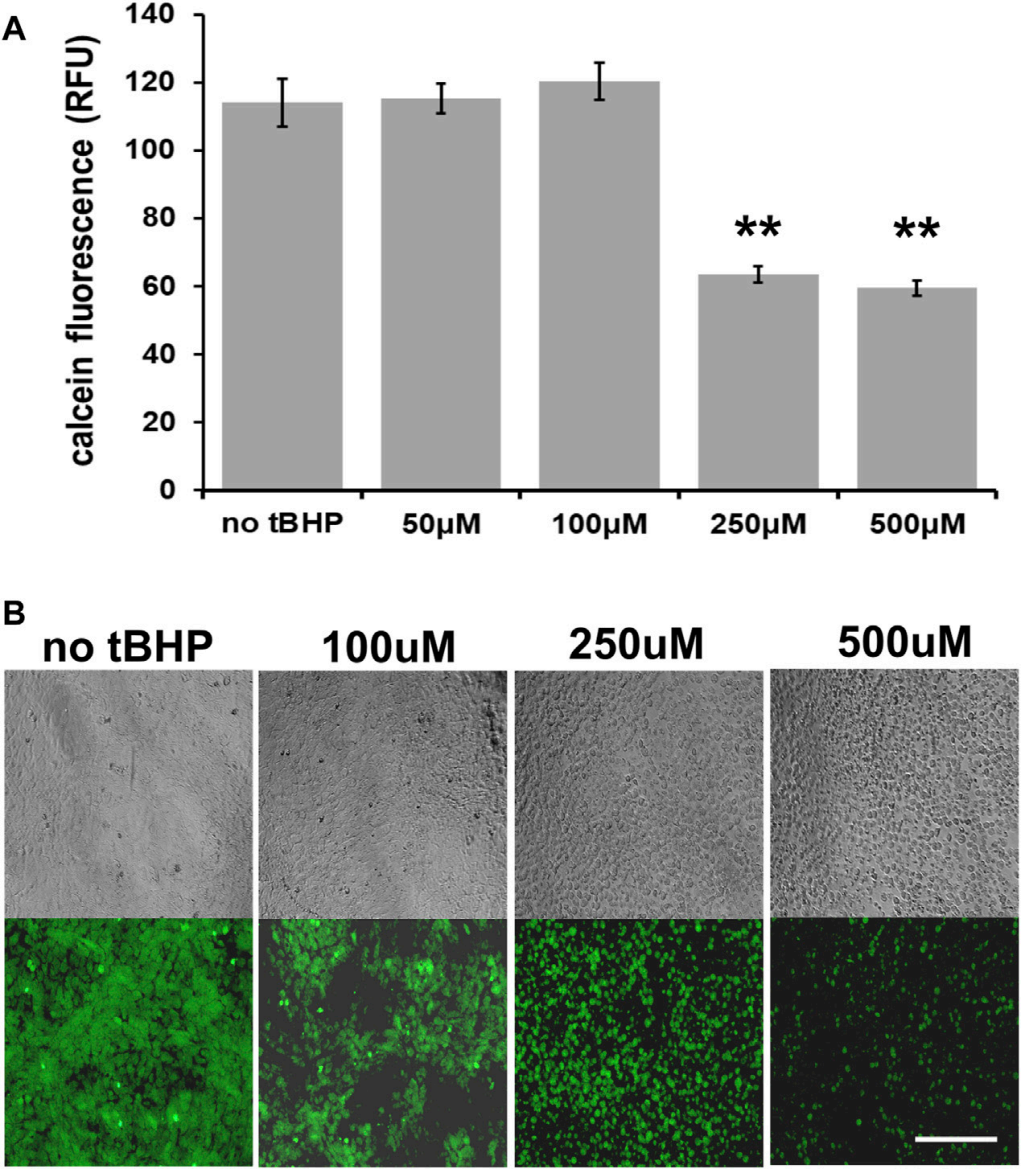
Sensitivity of hTERT-RPE cells to tert-butyl hydroperoxide (tBHP). **(A)** Calcein assay results of hTERT-RPE) cells exposed to different concentrations of tBHP (50, 100, 250, and 500 μM) for 24 h to establish a kill curve. Exposure of cells to 250 and 500 μM tBHP led to 44 and 47% reduction in calcein fluorescence, respectively. Calcein-AM assay data are an average of eight samples. One-way analysis of variance (ANOVA) analysis was performed to determine differences between groups. **p = ≤ 0.01 versus VC-tBHP. **(B)** Microscopic images of calcein-stained tBHP-exposed hTERT-RPE cells visually showing the tBHP concentration response. Top panel row—representative phase contrast images; bottom panel row—representative calcein fluorescence (488 nm/530 nm excitation/emission) images. Scale bar is 100 μm.

We first determined the optimal tBHP concentration to use for cytoprotection experiments. At confluence (100% cell density), 100 μM tBHP exhibits no noticeable loss of cell viability (as determined by a calcein fluorescence plate reader assay) while 250 and 500 μM decreases viability by 44 and 47%, respectively (Figure 1A). As a result, we used a high tBHP concentration of 500 μM in this study to determine cytoprotection provided by vitamin E components. Although high concentrations of tBHP (250–500 μM) reduced cellular esterase activity measured by calcein fluorescence, microscopic observation of calcein-AM loaded cells revealed that few cells became detached within 24 h after tBHP exposure and instead exhibited loss of cellular morphology, a common sign of cell death (Figure 1B) This microscopy approach allows a qualitative in addition to quantitative assessment of cell health unlike plate reader assays which offer only quantitative data.

### Cytoprotective Effects of the Main Vitamin E Components Against Oxidative Damage in hTERT-RPE Cells

**FIGURE 2:**
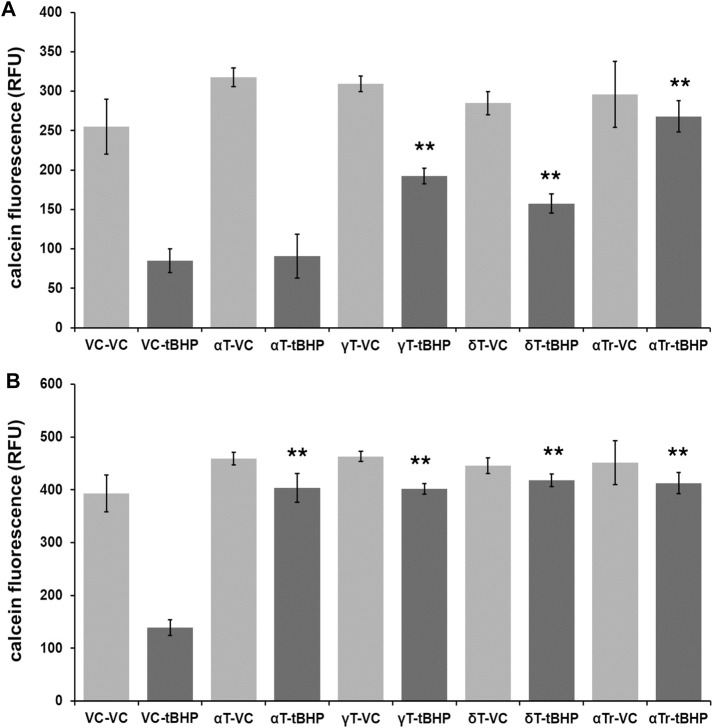
Viability of RPE cells exposed to tocopherols and αTr for either four or 24 h followed by exposure to tBHP (500 μM) for 24 h measured using the calcein-acetomethoxy ester (AM) assay. **(A)** Calcein-AM assay results of hTERT-REP cells exposed to 4-h of vitamin E components or vehicle control (VC) followed by 24-h tBHP exposure. Light gray bars are conditions with no tBHP while dark gray bars are conditions with 500 μM tBHP. The 4-h αT exposure offered no protection of hTERT-RPE cells against tBHP. **(B)** Calcein-AM assay results of hTERT-REP cells exposed to vitamin E components (or VC) for 24-h followed by 24-h tBHP exposure. Light gray bars are conditions with no tBHP while dark gray bars are conditions with 500 μM tBHP. At 24 h, all vitamin E components protected (≥85% protection) hTERT-RPE cells against tBHP. One-way ANOVA analysis with post hoc Bonferroni correction was performed to determine differences between groups. **p = ≤ 0.01 versus vehicle control (VC)-tBHP. Calcein assay data from each data set are from an average of four samples. Data for 4-h tocopherol and 24-h tocopherol groups were compared using a one-way ANOVA with Tukey’s post hoc test to determine whether tocopherol protection was time-dependent.

To determine if vitamin E components protect hTERT-RPE against severe (lethal) oxidative damage elicited by 500 μM tBHP and help maintain cell viability, we exposed hTERT-RPE cells to vitamin E components (αT, γT, δT and αTr—100 μM of each) for either four or 24 h, washed cells and then exposed cells to tBHP (500 μM) for an additional 24 h. After the 24-h tBHP insult, we carried out a calcein assay to measure cell viability. None of the tocopherols (α-, γ-, and δ) nor αTr exhibited any measurable toxicity at 100 μM (Figure 2).

The shorter 4-h tocopherol exposure time resulted in a differential degree of protection with γT and δT and αTr offering some protection (60, 50, and 90%, respectively) (Figure 2A). The 4-h αT exposure offered no protection against tBHP (Figure 2A). The longer 24-h tocopherol exposure time resulted 90% protection with all vitamers tested (Figure 2B). This suggests that the protective effect of αT against oxidative stress is time-dependent, whereas the other vitamers are not.

After 24-h exposure to tBHP, cells remained largely attached to plate wells making the calcein assay limited in determining the percentage of non-viable cells. As a result, we use microscopic methods to measure loss of cellular morphology and nuclear shrinkage (pyknotic nuclei) as surrogate markers of apoptotic cell death.

### Effects of Tocopherols and α-Tocotrienol on Actin Dynamics

The actin cytoskeleton is critical for the maintenance of cell morphology, development and motility. Since cellular morphology is significantly affected by cell death, including apoptosis (Laster and Mackenzie, 1996; Levee et al., 1996), we labeled filamentous actin with fluorescently labeled phalloidin, which binds with high affinity to filamentous but not globular actin. This labeling allowed us to easily visualize changes in cell morphology that accompany cell death.

**FIGURE 3:**
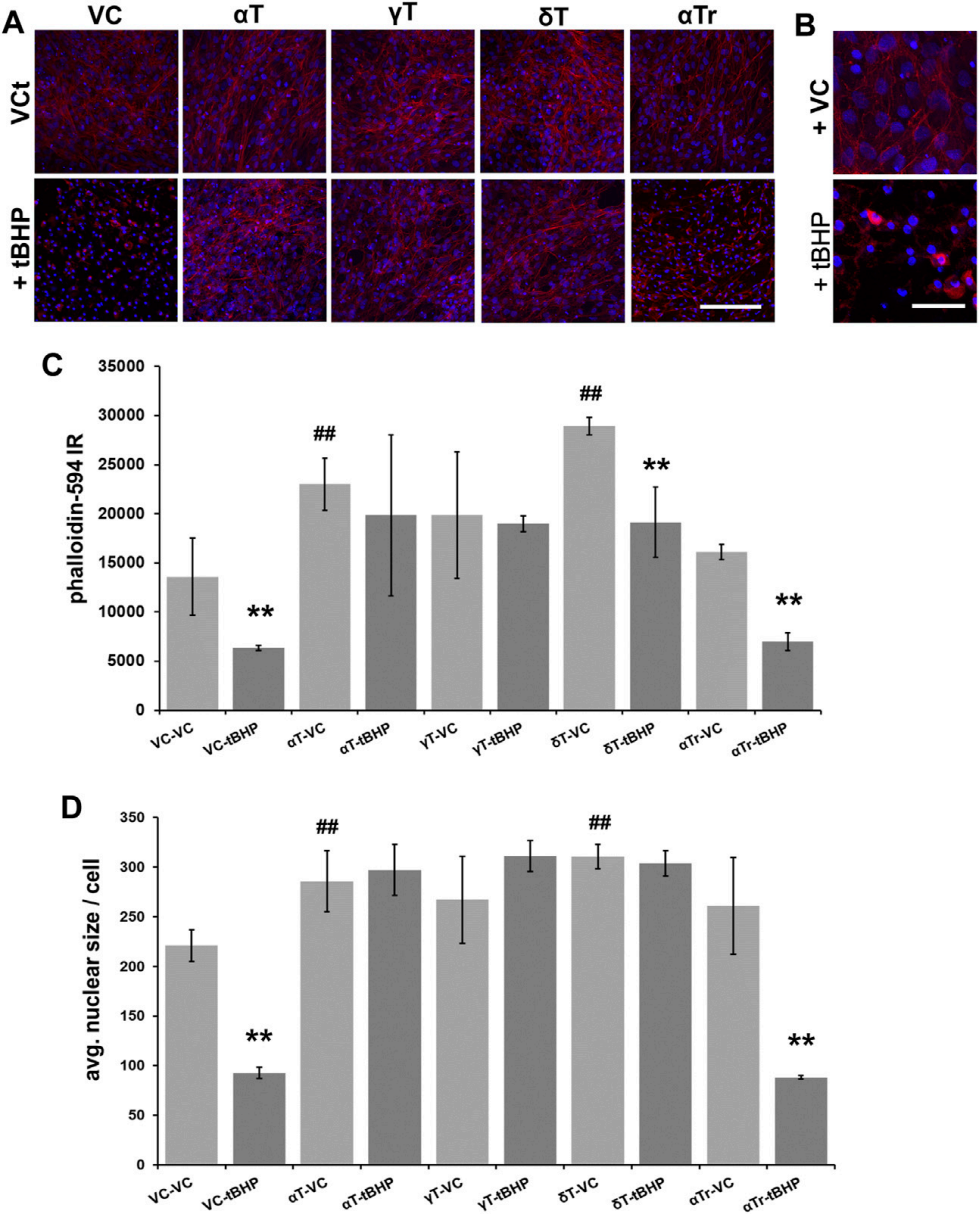
Effect of 24-h αT, γT and δT and αTr exposure on RPE actin dynamics after subsequent tBHP-induced oxidative stress. RPE cells were exposed to a vehicle control (VC) αT, γT or δT or αTr for 24 h prior to tBHP. **(A)** tBHP reduced phalloidin fluorescence (red) and nuclear area (DAPI, blue fluorescence). A reduction in filamentous actin (phalloidin (red) fluorescence) is suggestive of actin destabilization observed in cell death resembling apoptosis. Nuclear size (blue) is reduced during cell death, particularly during apoptosis. Images were acquired at 40X magnification; scale bar is 100 μm. **(B)** A close up image of tBHP-mediated reduction in filamentous actin labeling by phalloidin and reduction in nucleus size caused by tBHP exposure. Scale bar is 25 μm. **(C)** Graphical data showing that exposure of cells to αT and γT, and to a lesser extent δT, prevented this effect in hTERT-RPE cells. αTr, however, could not prevent the reduction of phalloidin staining and nuclear size. **(D)** Exposure of cells to αT and δT led to an increase in nuclear size. Exposure of cells to tBHP led to a reduction in nucleus size indicative of pyknosis. The exposure of cells to αT, γT and δT prevented the tBHP-mediated reduction in nuclear size, while exposure of cells to αTr did not. **p ≤ 0.01 with tBHP versus the same condition without tBHP; ^##^ *p* ≤ 0.01 with tocopherol versus without tocopherol. Microfluorimetric analyses are from an average of three images. One-way ANOVA analysis with post hoc Bonferroni correction was performed to determine differences between groups.

Pre-exposure of cells to αT and δT (100 μM of each) for 24 h increased phalloidin labeling of filamentous actin, suggesting that actin was stabilized by these tocopherols (Figure 3A). Gamma-tocopherol appeared to increase phalloidin-594 fluorescence, but it failed to reach statistical significance (Figures 3A,C). Pre-exposure of cells to γT and δT prevented tBHP-mediated actin destabilization, whereas αTr did not. Pre-exposure to αTr resulted in a decrease in phalloidin labeling in cells to the degree observed in controls, suggesting that αTr may preserve some measures of cell viability (cellular esterase activity (Figure 1), but not maintenance of the actin cytoskeleton. (Figure 3). These results also suggest that there are differences in the vitamers tested in their ability to protect RPE from oxidative damage.

To determine if αT, γT, δT and αTr prevent tBHP-mediated formation of pyknotic nuclei, we measured DAPI-stained nuclei to measure the average nuclear size (Figures 3A,D). None of the vitamin E components alone affected nuclear size or number. Exposure to tBHP led to a 40% decrease in nuclear size indicative of the nuclear shrinkage (pyknosis) observed in later stages of cell death resembling that of apoptosis (Figures 3A,B). All three tocopherols (αT, γT and δT), but not αTr, prevented tBHP-mediated nuclear shrinkage (pyknosis).

### Effects of αT and γT and Sublethal Oxidative Stress on Nrf2 and SOD2 Immunoreactivity

After establishing sublethal versus lethal concentrations of tBHP for hTERT-RPE cells, we determined what effect αT and γT and/or sublethal tBHP exposures had on the expression of nuclear factor erythroid 2-related factor 2 (Nrf2) and superoxide dismutase 2 (SOD2). We decided to focus on αT and γT because 1) much more is known about αT than any other form of tocopherol, and it is generally considered the prototypical tocopherol and 2) γT is also relatively well studied and it is the most abundant tocopherol from food sources. Furthermore, a few studies have compared the activity of αT and γT thereby determining that there are differences in their activity.

Nrf2 is a redox sensitive transcription factor that regulates the expression of genes involved in the cellular antioxidant response while Sod2 is a mitochondrial antioxidant enzyme that is critical for maintaining redox balance in cells (Murakami et al., 1998; Venugopal and Jaiswal, 1998; Itoh et al., 1999; Melov et al., 1999). These proteins were chosen as they should be useful markers for the cellular response against oxidative stress.

**FIGURE 4:**
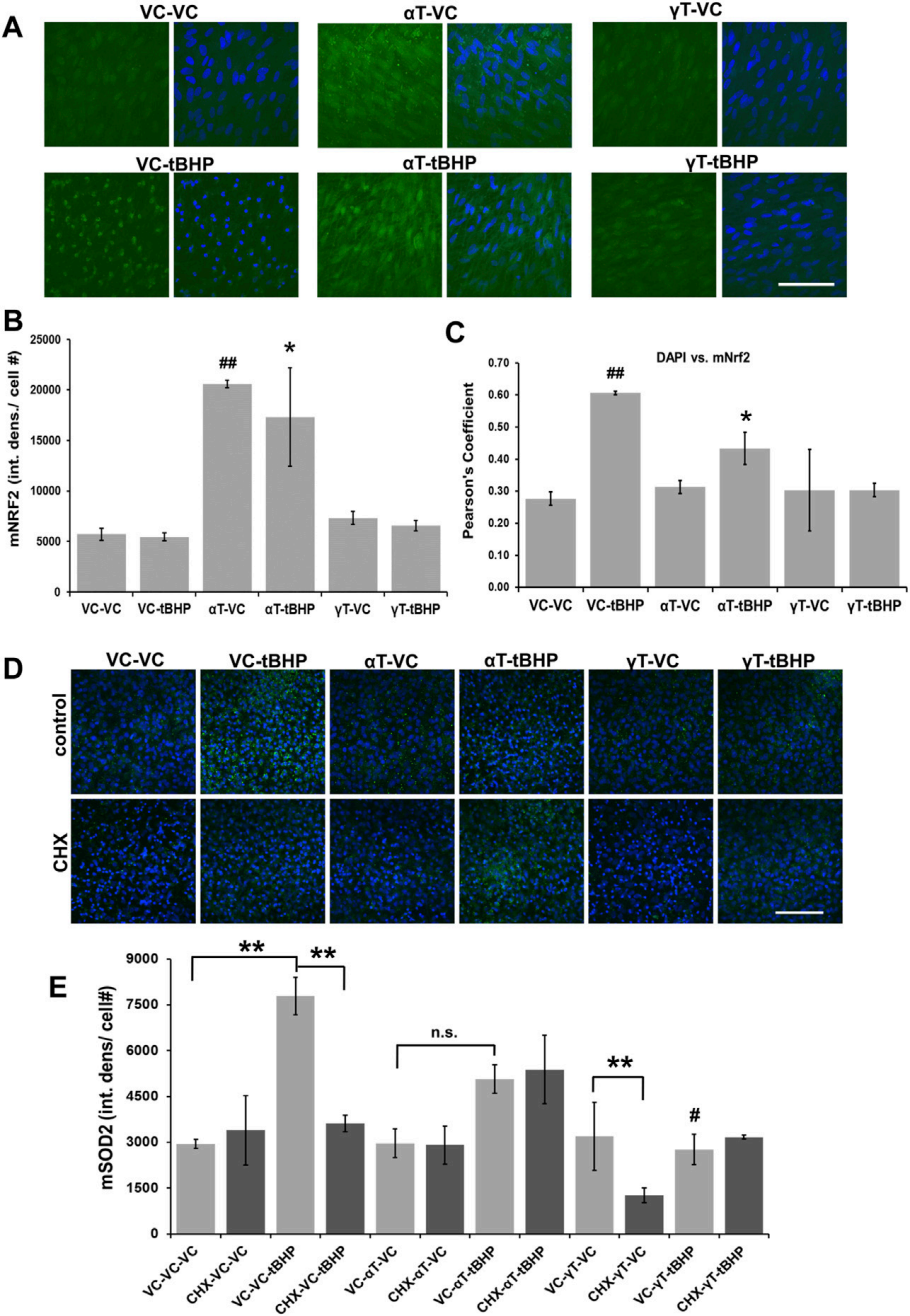
Effect of αT, γT and Sublethal Oxidative Stress on Antioxidant Nrf2 and Sod2 Protein Expression. **(A)** The image panel shows Nrf2 IR (green) and the nucleus (blue) in the presence or absence of αT or γT with or without tBHP. Images are 63x magnification and the scale bar is 50 μm. **(B)** Quantitative graphical data calculated from images revealed that αT induced Nrf2 expression (Nrf2 IR) by 3.8-fold. Furthermore, oxidative stress by tBHP had no effect on αT-mediated increase in Nrf2 IR. **(C)** Colocalization analysis between Nrf2 IR and DAPI fluorescence reveals that tBHP, but not αT nor γT, leads to Nrf2-DAPI colocalization (r^2^ = 0.60). The presence of prevented this tBHP-mediated nuclear translocation of nrf2 and resulting Nrf2-DAPI colocalization. Microfluorimetric analyses are from an average of three images. *p = ≤0.05 versus VC-tBHP, *^##^ *p* = ≤0.01 versus VC-VC as determined by one-way ANOVA analysis with Bonferroni post hoc test. **(D)** The image panel shows SOD2 IR (green) and the nucleus (blue) in the presence or absence of CHX as well as αT or γT with or without tBHP. Images are 40x magnification and the scale bar is 100 μm. **(E)** Quantitative data calculated from images revealed that exposure of cells to either αT or γT had no effect of SOD2 IR, while exposure to sublethal (100 μM) tBHP led to a 2.6-fold increase in SOD2 IR. Exposure of cells to αT does not significantly reduce tBHP-mediated induction of Sod2. Exposure of cells to γT led to a 64% reduction in tBHP-mediated induction of SOD2. Exposure of cells to CHX had no effect on Sod2 IR, but CHX with γT led to a 60% decrease in Sod2 IR. CHX reversed tBHP-mediated induction of Sod2. CHX had no effect on αT’s nor γT’s ability to reduce the induction of Sod2 mediated by tBHP. **p = ≤0.01; n.s.—not significant; ^#^ *p* = ≤0.01 versus VC-αT-tBHP as determined by one-way ANOVA analysis with Bonferroni post hoc test.

Exposure of cells to αT, but not γT, led to a 4-fold increase in Nrf2 IR while exposure to tBHP had no effect on the amplitude of Nrf2 IR (Figures 4A,B). Exposure of cells to αT followed by tBHP led to a 3.5-fold increase in Nrf2 IR while exposure to γT followed by tBHP had no effect on Nrf2 IR similar to that of tBHP or γT exposures alone (Figures 4A,B).

To determine what effect αT, γT and/or tBHP had on NRf2 nuclear translocation, we measured the colocalization between DAPI DNA stain in the nucleus and Nrf2 IR (Figure 4C). Although exposure to tBHP had no effect on the amplitude of Nrf2 IR, tBHP exposure did increase the amount of Nrf2 IR in the nucleus as determined by an increase in DAPI-Nrf2 colocalization and Pearson’s correlation test (r^2^ = 0.61 (VC-tBHP) vs. 0.28 (VC-NT)) (Figure 4C). This suggests that a 24-h period of oxidative stress leads to the nuclear translocation of Nrf2, but not an increase in its expression levels. Alpha-tocopherol exposure itself for 24 h led to the upregulation of Nrf2 expression but not Nrf2 nuclear translocation. αT, but not γT, reduced the tBHP-mediated nuclear translocation of Nrf2 by 27% further highlighting a differential effect between αT and γT on Nrf2 activity.

Exposure of cells to either αT or γT had no effect of SOD2 IR, suggesting that they, themselves, cannot induce SOD2 gene expression (Figures 4D,E). Exposure of cells to sublethal (100 μM) tBHP for 24 h led to a 2.6-fold increase in SOD2 IR, which was expected as others have shown that the Sod2 gene is induced by oxidative stress (Murakami et al., 1998; Melov et al., 1999). Exposure of cells to αT does not significantly reduce tBHP-mediated induction of Sod2, but it shows a trend toward a decrease (*p* = 0.22) (Figure 4E). Exposure of cells to γT led to a 64% reduction in tBHP-mediated induction of SOD2. Together, this suggests that γT is superior to αT in reducing the oxidative stress elicited by the level of tBHP needed for Sod2 gene induction.

To determine if synthesis of new proteins is required for tocopherol-mediated cytoprotection, we pre-treated hTERT-RPE cells with the protein synthesis inhibitor, cycloheximide, 1 h prior to tocopherol exposure and 24 h prior to tBHP exposure.

Exposure of cells to CHX, with or without αT, also had no effect on Sod2 IR, CHX together with γT led to a 60% decrease in Sod2 IR (Figure 4E). CHX completely reversed the tBHP-mediated induction of Sod2, suggesting that protein synthesis is required for tBHP induction of Sod2 (Figure 4E). CHX had no effect on αT’s nor γT’s ability to reduce the induction of Sod2 mediated by tBHP (Figure 4E), indicating that the mechanism by which these tocopherols reduce tbHP-mediated Sod2 is not dependent upon protein synthesis.

### Effects of αT and γT on ZO-1 Under Conditions of Oxidative Stress

RPE barrier function (retinal-blood barrier) is compromised in AMD which prompted us to detect and measure the actin-interacting tight junction protein, ZO-1, as a measure of possible barrier integrity (Penfold et al., 1990). ZO-1 is a protein critical for tight junction formation and reports from others (Pu et al., 2005; Yang et al., 2015), and bioinformatics analysis, suggests that the ZO-1 gene may be regulated by oxidative stress.

We determined whether αT and γT and/or sublethal tBHP exposures had on the expression of zona occludens 1 (ZO-1) a critical component of tight junctions in endothelial and epithelial cells, such as RPE (Pu et al., 2005; Yang et al., 2015). ZO-1 directly interacts with multiple proteins in tight junctions and with the actin cytoskeleton (Nybom and Magnusson, 1996; Itoh et al., 1997).

**FIGURE 5:**
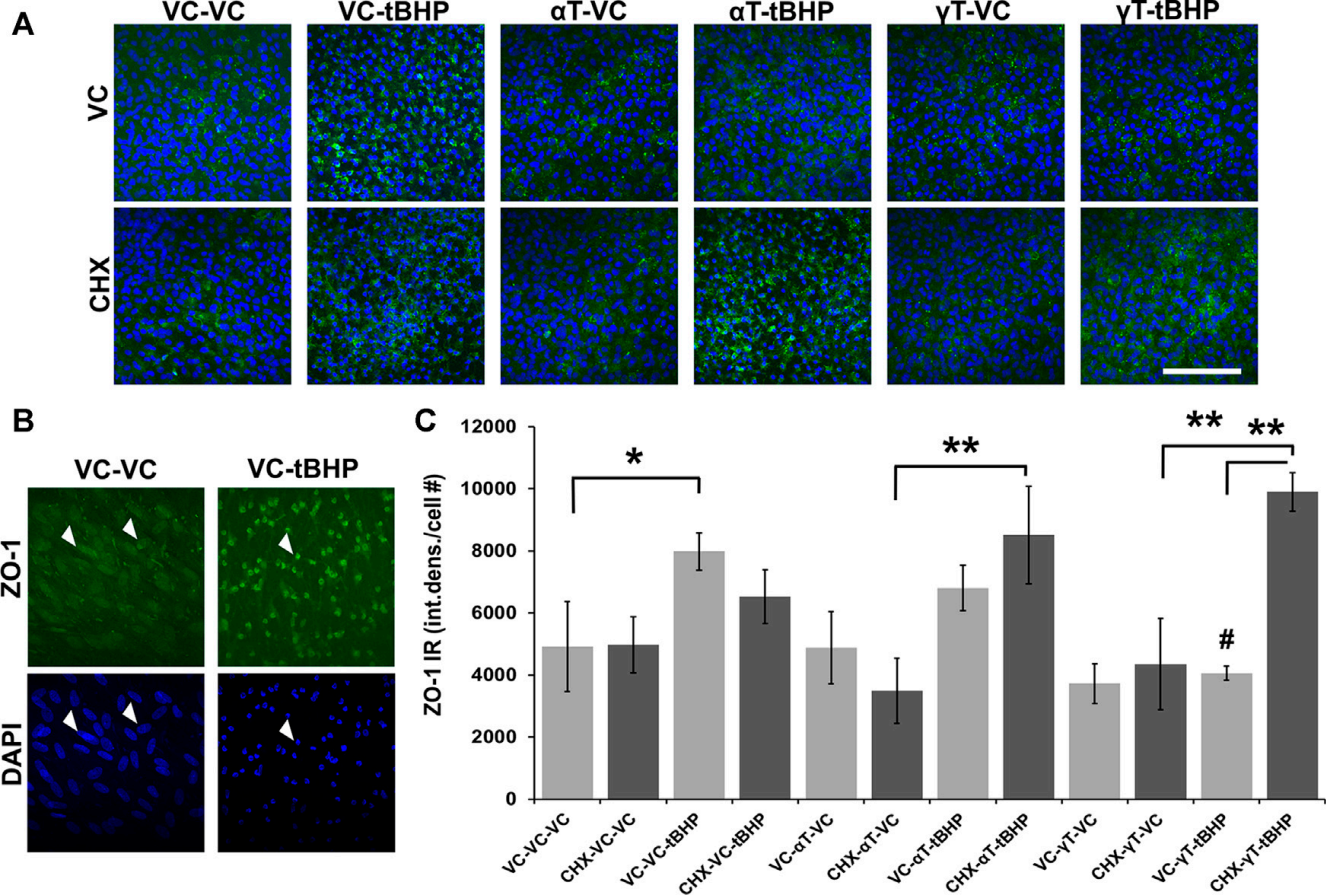
Effect of αT, γT, Oxidative Stress and Protein Synthesis on ZO-1 Expression. **(A)** The image panel shows ZO-1 IR (green) and the nucleus (blue) in the presence or absence of αT or γT, with or without tBHP and with or without CHX. Images are 63x magnification and the scale bar is 50 μm. **(B)** Optically magnified images demonstrating condensed ZO-1 IR (white arrows) and nuclear shrinkage (white arrows) observed under conditions of oxidative stress. **(C)** Quantitative graphical data calculated from images revealed that tBHP alone, but neither αT nor γT alone, induced ZO-1 expression (ZO-1 IR, green) by 2.6-fold. The presence of αT reduced the effect of tBHP on ZO-1 induction, but it failed to reach statistical significance. The presence of γT, however, completely prevented tBHP’s effect on ZO-1 induction. Furthermore, inhibition of protein synthesis with CHX did not significantly affect tBHP-mediated ZO-1 induction nor αT-tBHP’s effect on ZO-1 IR. CHX, however, completely prevented γT’s reversal of tBHP-mediated ZO-1 induction. Microfluorimetric analyses are from an average of three images. *p = ≤0.05, **p = ≤ 0.01 with tBHP versus the same condition without tBHP as determined by one-way ANOVA analysis with Bonferroni post hoc test.

Exposure of cells to either αT or γT had no effect of ZO-1 IR (Figures 5A,B). Exposure of cells to sublethal (100 μM) tBHP for 24 h led to a 63% increase in ZO-1 IR, particularly in the nucleus (Figure 5C). Nuclear localization of ZO-1 has been reported elsewhere and has been shown to play a role in multiple cellular functions (Zhong et al., 2012; Guo et al., 2017; Lesage et al., 2017). Similarly, exposure of cells to αT does not significantly reduce tBHP-mediated induction of ZO-1, but it shows a trend toward a decrease (Figure 5C). Exposure of cells to γT led to a 50% reduction in tBHP-mediated induction of ZO-1 (Figure 5C). This suggests that, unlike αT, γT prevents the effects of tBHP on ZO-1 expression (Figure 5C), thus is more potent than αT in maintaining RPE function.

Exposure of cells to CHX, with or without αT or γT, also had no effect on ZO-1 IR. Addition of the protein synthesis inhibitor, CHX, does not significantly reduce tBHP-mediated induction of ZO-1, but it shows a trend toward a decrease (Figure 5C). This suggests that protein synthesis may be a factor in the induction of ZO-1 expression elicited by oxidative stress (tBHP). CHX and αT prior to tBHP exposure, however, had no effect on ZO-1 IR, while CHX completely reversed the ability of γT to reduce tBHP-mediated increase in ZO-1 IR (Figure 5C). This suggests that, unlike αT, γT prevents the effects of tBHP on ZO-1 expression and these effects require protein synthesis (Figure 5C).

### Effects of αT and γT on Nuclear Shrinkage Under Conditions of Severe Oxidative Stress

**FIGURE 6:**
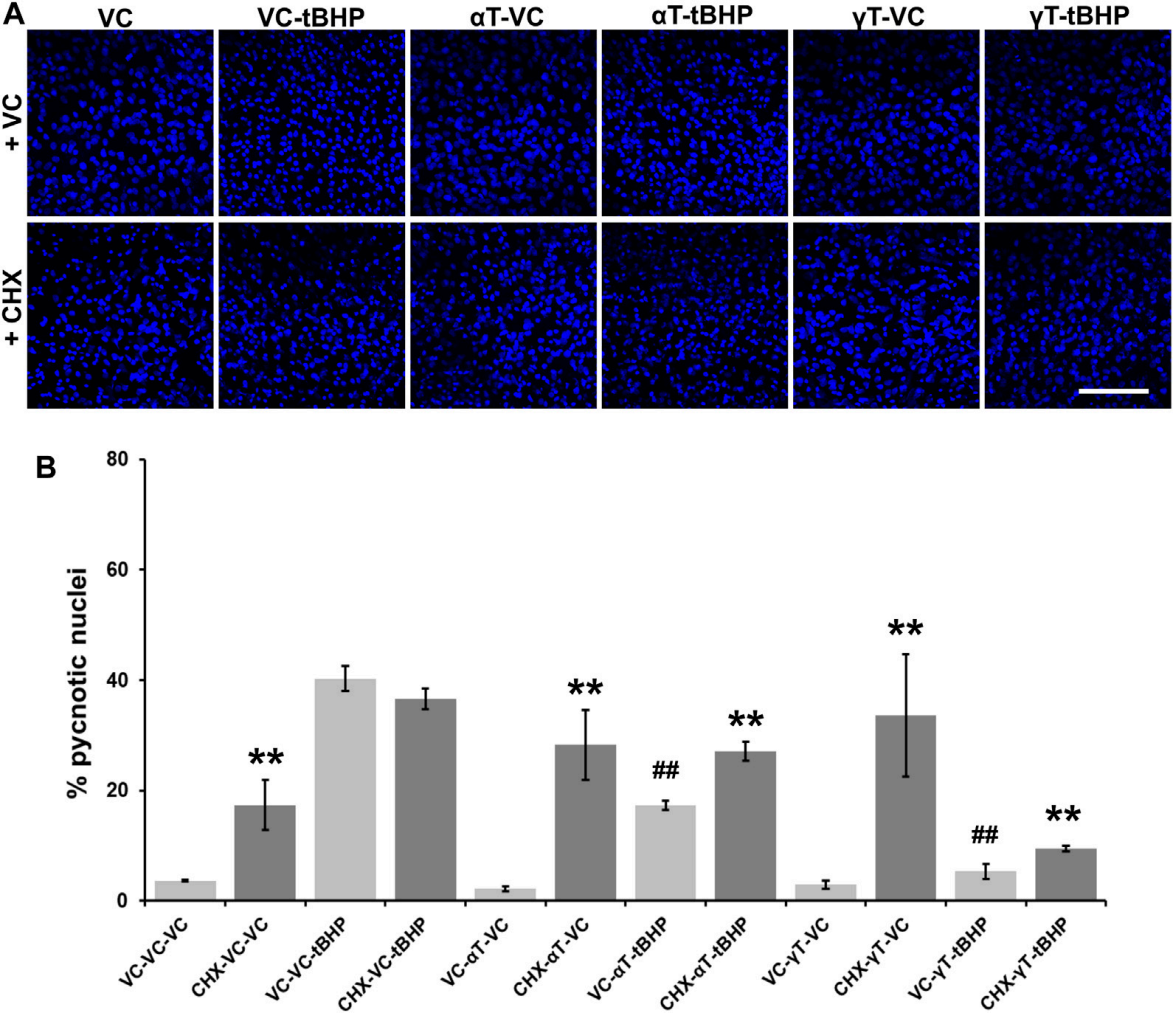
Effect of CHX and long-term (24-h) tocopherol exposure of hTERT-RPE cells on tBHP-mediated cytotoxicity. hTERT-RPE cells were exposed to CHX for 1 h followed by αT or γT for 24 h prior to tBHP. **(A)** Image panels showing DAPI labeling of nuclei (blue) in cells exposed to CHX, αT or γT and tBHP. Top row represents conditions without CHX while the bottom row represents conditions with CHX. Nucleus area (blue fluorescence) is reduced during cell death by a process resembling apoptosis. Images acquired at 40x magnification; scale bar is 100 μm. **(B)** Measurement of pyknotic nuclei, nuclei that are half the size of average normal nuclei and not merely reduced in size, was carried out on CHX-, tocopherol- and tBHP-exposed cells. Exposure of cells to CHX (dark bars) alone increases the percentage of pyknotic nuclei in the cell population from 4% in control cells (light bars) to 17%. Exposure of cells to tBHP led to an increase in the percentage of pyknotic nuclei from 4 to 40%. Exposure of cells to either tocopherol alone had no effect on the percentage of pyknotic nuclei, but exposure to CHX and tocopherols led to an increase in the percentage of pyknotic nuclei from 2 to 28% for αT and 3–34% for γT. The presence of αT prior to tBHP insult reduced the percentage of pyknotic nuclei by 57% compared to cells treated with tBHP alone. Inhibition of protein synthesis by CHX partially reversed this αT protection by 47%. The presence of γT prior to tBHP insult reduced the percentage of pyknotic nuclei by 87% compared to cells treated with tBHP alone. Inhibition of protein synthesis by CHX partially reversed this γT protection by 44%. Microfluorimetric analyses are from an average of three images. **p ≤ 0.01 with CHX versus the same condition without CHX, ^##^ *p* ≤ 0.01 with tBHP versus the same condition without tBHP as determined by one-way ANOVA analysis with Bonferroni post hoc test.

Quantitation of pyknotic nuclei, nuclei that appear to be half or less the size of normal nuclei, were counted and expressed as a percentage of the total (Figures 6A,B). Exposure of cells to tBHP led to a 37% increase in nuclear pyknosis compared to controls (Figure 6B). Exposure of cells to αT or γT had no effect on baseline nuclear pyknosis. Exposure of cells to αT or γT decrease tBHP-mediated nuclear pyknosis by 55 and 89%, respectively (Figure 6B).

Pretreatment of cells with CHX, itself, lead to a 4.3-fold increase in nuclear pyknosis compared to control not treated with CHX (13% overall increase) (Figure 6B). CHX treatment prior to tBHP, led to a 10% decrease in nuclear pyknosis compared to tBHP alone (5% decrease overall), suggesting that protein synthesis is required, in part, for nuclear pyknosis. Pretreatment of cells with CHX prior to αT led to an 18-fold increase the nuclear pyknosis compared to cells exposed to αT alone (19% overall increase in nuclear pyknosis (Figure 6B). Exposure of cells to CHX prior to αT and tBHP led to a 1.7-fold increase in nuclear pyknosis (13% overall increase) compared to αT and tBHP without CHX pre-exposure, suggesting that some protein synthesis is required for αT to reduce tBHP-mediated nuclear shrinkage.

Pretreatment of cells with CHX prior to γT led to an 11-fold increase the nuclear pyknosis compared to cells exposed to γT alone (31% overall increase in nuclear pyknosis) (Figures 6A,B). Exposure of cells to CHX prior to γT and tBHP led to a 2-fold increase in nuclear pyknosis (5% overall increase) compared to γT and tBHP without CHX pre-exposure, suggesting that some protein synthesis is required for γT to reduce tBHP-mediated cell death (Figure 6).

## Discussion

Here, we determined that specific vitamin E components exhibit unique cytoprotective properties in immortalized human RPE cells. We used supraphysiological concentrations of γT, δT and αTr to ascertain whether they exhibit similar cytoprotective activity as αT. In addition, we determined whether short-term versus longer-term exposure to tocopherols leads to similar degree of cytoprotection against oxidative stress and cell death. Both γT, δT and αTr require less time than αT to protect cells against tBHP, suggesting that αT acts through a different mechanism of action than direct antioxidant activity to exert its full protective effects. We focused primarily on αT and γT because there are more published studies on these two tocopherols than other vitamin E components and they are metabolized differently (Azzi, 2018; Uchida et al., 2018). Our results suggest that αT and γT exhibit different kinetics and/or potencies for antioxidant activity, different effects on Nrf2 expression and different effects on actin stability, Sod2 and ZO-1 expression meditated by oxidative stress. As a result of our data, the selection of specific tocopherol vitamers as therapeutic antioxidants may need to be considered, particularly γT, due to its different pharmacokinetic and pharmacodynamic properties than αT.

To our knowledge, this study is the first measuring the comparative kinetics and cytoprotective efficacy between αT and γT against oxidative damage, the expression and subcellular localization of antioxidant and structural proteins, and the requirement for protein synthesis for these effects in RPE cells.

Many plant-based vitamin E sources contain predominantly γT and lesser concentrations of other tocopherols (Jiang et al., 2001; Szewczyk et al., 2021). Since North American diets contain more γT than other tocopherols, higher levels of γT tocopherol are ingested, but it is more rapidly eliminated from the body (in bile). αT, on the other hand, persists at higher plasma concentrations due to much greater retention in the body. There are conflicting reports about which tocopherol form exhibits the most potent antioxidant activity (Kamal-Eldin and Appelqvist, 1996). Based on literature, the upper physiologic concentration of vitamin E in mammals is in the low micromolar range (<25 μM) (Arigony et al., 2013; Jamro et al., 2019). Based on this we chose to use 100uM tocopherols and tocotrienols, as this concentration is up to 10 times higher than concentrations achieved through a normal diet but could still be achieved using dietary supplementation.

We selected a short (4-h) tocopherol exposure time based on the assumption that it would not allow for as much cellular uptake through tocopherol transport proteins as the 24-h exposure period. Therefore, the shorter 4-h tocopherol exposure period was a better approach for determining the direct antioxidant effect while the 24-h period was a better approach for looking at cellular uptake and subsequent cell signaling, in addition to direct antioxidant properties.

Our rationale for selecting the measurement of actin, nuclei, Nrf2, Sod2 and ZO-1 is based on numerous studies where the detection and measurement of these proteins serve to assess the functional integrity (actin and ZO-1) and cellular response to oxidative stress (Nrf2 and Sod2) and viability (actin and nuclear staining) of epithelial cells (Penfold et al., 1990; Murakami et al., 1998; Venugopal and Jaiswal, 1998; Itoh et al., 1999; Melov et al., 1999; Pu et al., 2005; Yang et al., 2015).

To assess cell death in RPE in response to tBHP, we labeled actin filaments with fluorescently tagged phalloidin. Phalloidin is a cell permeable phallotoxin from the death cap mushroom that binds with high affinity to filamentous actin, but not depolymerized or monomeric actin (Cooper, 1987). During apoptotic cell death, some components of the cytoskeleton, including actin, have been shown to become destabilized or relocate to the periphery of the cell (Reed, 1995). As a result, hTERT-RPE cells exposed to tBHP exhibit reduced filamentous actin and phalloidin labeling. DAPI labels DNA in cells and during apoptotic cell death, nuclear condensation occurs. During this process, DNA migrates to the periphery of the nucleus near the nuclear envelope as part of a process called pyknosis (Reed, 1995). hTERT-RPE cells exposed to tBHP resulted in pyknotic nuclei. Since we did not measure caspase activation or other direct apoptotic markers, we could not prove that cell death was carried out by an apoptotic process. In this study, however, we were more interested in observing and measuring cellular morphology than a specific apoptotic process.

Since Nrf2 is a major regulator of the cellular antioxidant response, we decided to measure the effect of tBHP and tocopherols on its expression. We predicted that tBHP, and not tocopherols themselves, would increase Nrf2 expression. Much to our surprise, however, tBHP had no effect on Nrf2 expression but αT, not γT, did. The only effect of tBHP we were able to observe was the relative increase in nuclear Nrf2 IR compared to other treatments. Our results indicate that αT itself can upregulate Nrf2 expression, thereby potentially priming the RPE cells for a more effective antioxidant response. Interestingly, αT, but not γT, was able to reduce tBHP-mediated Nrf2 nuclear translocation suggesting that αT ang γT exhibit differential effects on Nrf2 activity.

Sod2 is an important antioxidant enzyme located in mitochondria, a common source of free radical formation (Murakami et al., 1998; Melov et al., 1999). We determine whether tBHP induces Sod2 expression because it has been shown in multiple studies to become induced in response to oxidative stress (Murakami et al., 1998; Melov et al., 1999). We also determined whether tocopherols could affect Sod2 expression. As expected, tBHP increased Sod2 IR, but neither αT nor γT had an effect on Sod2 IR. In fact, γT, and to a lesser extent, αT, decreased Sod2 IR elicited by tBHP. Since inhibiting protein synthesis with CHX didn’t change the inhibitory effect of αT or γT on tBHP-mediate-Sod2 expression, we conclude that these effects of αT and γT may not require the synthesis of new protein.

We determined whether tBHP, αT or γT or a combination of tocopherols and tBHP had an effect on the expression of the tight junction and actin-binding protein, ZO-1. We reasoned that a reduction or redistribution of ZO-1 may have significant negative ramifications for RPE tissue integrity and barrier function while an increase in ZO-1 IR would represent improvement in RPE function. Both tBHP and αT, alone, led to an increase in ZO-1 IR and the combination of both led to an additive increase in ZO-1. ZO-1 IR in these cells was predominantly nuclear and not located at the plasma membrane where one would expect to see it. Several reports have identified nuclear localization of ZO-1 which corroborate our findings. For example, in lung and breast tumor cells, ZO-1 has been observed in cytonuclear compartments and is involved in NFκB signaling (Lesage et al., 2017). The HIV Tat proteins has been shown to alter the expression pattern of ZO-1 in murine brain endothelial cells (Pu et al., 2005) and to cause nuclear localization of ZO-1 in human brain vascular endothelial cells (Zhong et al., 2012). The observation that ZO-1 is expressed on the cell surface and in the cytoplasmic and nuclear regions is interesting, but the ramifications for these different expression patterns are not clear.

The upregulation of ZO-1 by αT, tBHP and both αT and tBHP suggest that, 1) αT may help protect barrier function by creating more cellular ZO-1 protein, 2) cells may respond to oxidative stress by upregulating ZO-1 to help maintain proper barrier and, 3) the presence of both produce an additive effect. The fact that γT does not have the same effect on ZO-1 expression indicates that the two tocopherols exhibit different activities or interact with different cellular processes.

Cycloheximide inhibits protein synthesis by preventing the translocation of transfer RNA in the ribosomal active site. To determine if protein synthesis is required for tocopherol’s protective effects, we exposed cells to CHX 1 h before the addition of tocopherols so that the inhibition of protein synthesis would occur before, or in the early stages of, tocopherol-mediated signal transduction. The presence of CHX should not have any effect on tocopherols direct antioxidant activities. CHX can be toxic to some cells at concentrations needed to block protein synthesis, so we removed the CHX within 1 h, when the tocopherols were added, to allow cells to recover from its toxic effects prior to exposing cells to tBHP. CHX and tBHP toxicity are not additive; this suggests that, as expected, the mechanism of action of CHX and tBHP are mutually exclusive.

Future studies need to be carried out to determine whether αT, γT and δT can affect RPE function, particularly the secretion of neurotrophic and protective factors, uptake of shed photoreceptor outer segments and barrier function as determined by transepithelial resistance. In addition, several questions regarding the *in vitro* and *in vivo* kinetics and potency of different tocopherol and tocotrienols must be answered. It is also reasonable to assume that specific tocopherol-tocotrienol combination formulations, together with localized delivery methods, in lieu of systemic administration, may provide great therapeutic potential in diseases where oxidative stress is a major contributor.

## Data Availability

The original contributions presented in the study are included in the article/Supplementary Material, further inquiries can be directed to the corresponding author.

## Abbreviations

ANOVA, analysis of variance; αT, γT, δT, alpha tocopherol, gamma tocopherol and delta tocopherol, respectively; αTr, alpha tocotrienol; AMD, age-related macular degeneration; CHX, cycloheximide; DNA, deoxyribonucleic acid; DAPI, 4′,6-diamidino-2-phenylindole; hTERT, human telomerase reverse transcriptase; Nrf2, nuclear factor erythroid 2-related factor 2; RPE, retinal pigment epithelium; Sod2, superoxide dismutase 2; tBHP, tert-butyl hydroperoxide; VC, vehicle control; ZO-1, zona occludens.
